# Protocol for a multicenter randomized controlled trial comparing a non-opioid prescription to the standard of care for pain control following arthroscopic knee and shoulder surgery

**DOI:** 10.1186/s12891-021-04354-x

**Published:** 2021-05-22

**Authors:** Aaron Gazendam, Seper Ekhtiari, Nolan S. Horner, Nicole Simunovic, Andrew Duong, Darren de Sa, Devin Peterson, Matthew Denkers, Vickas Khanna, Anthony Adili, Jaydeep Moro, Moin Khan, Olufemi R. Ayeni, The NO PAin Investigators, Aaron Gazendam, Seper Ekhtiari, Nolan S. Horner, Nicole Simunovic, Andrew Duong, Darren de Sa, Devin Peterson, Matthew Denkers, Vickas Khanna, Anthony Adili, Jaydeep Moro, Moin Khan, Olufemi R. Ayeni

**Affiliations:** Department of Surgery, Division of Orthopaedic Surgery, McMaster University, 1200 Main St West, 4E15, Hamilton, ON L8N 3Z5 Canada

**Keywords:** Arthroscopy, Opioid sparing, Nonopioid, Randomized controlled trial

## Abstract

**Background:**

Opioids continue to be the analgesic of choice for postoperative pain control following arthroscopic knee and shoulder surgery. Despite their widespread use, there are limited evidence-based clinical practice guidelines for postoperative opioid prescribing. The Non-Opioid Prescriptions after Arthroscopic Surgery in Canada (NO PAin) Trial is a randomized controlled trial (RCT) designed to determine whether a non-opioid analgesia approach to postoperative pain, compared to usual care, reduces oral morphine equivalents (OME) consumed in patients undergoing outpatient knee and shoulder arthroscopy.

**Methods:**

This is a multi-centre, RCT with a target sample size of 200 patients. Adult (18+ years of age) patients undergoing outpatient knee and shoulder arthroscopy will be randomized to a non-opioid postoperative protocol (intervention) or the current standard of care (control). The intervention will consist of a standardized non-opioid analgesic prescription, a limited rescue opioid prescription, and a patient education infographic. The control is defined as the treating surgeons’ pre-trial postoperative analgesic regimen. Exclusion criteria include chronic opioid use, concomitant open surgery, contraindications to the prescribed analgesics or ongoing workers compensation/litigation. The primary outcome is OMEs consumed at 6 weeks postoperatively. Secondary outcomes will include patient-reported pain and satisfaction, quantity of OMEs prescribed, number of opioid refills, and any adverse events up to 6 weeks postoperatively. Utilizing the intention to treat principle for all analyses, independent samples t-test and presented with a *p*-value as well as a mean difference (MD) with 95% confidence intervals (CIs) will be performed for primary and secondary outcomes.

**Discussion:**

The ongoing opioid epidemic and overprescribing of opioids in orthopaedics serve as the rationale for this trial. There is a lack of evidence upon which to develop post-operative pain management guidelines for patients undergoing arthroscopic surgery. A prospective evaluation of this relatively inexpensive intervention will demonstrate whether an explicit effort to reduce the number of opioids prescribed results in a reduction in the amount of opioids consumed and help to inform future studies and guidelines.

**Trial registration:**

The NO PAin trial has been prospectively registered with clinicaltrials.gov (NCT04566250).

**Supplementary Information:**

The online version contains supplementary material available at 10.1186/s12891-021-04354-x.

## Background

Canada has the second highest per-capita opioid use in the world [1]. The Canadian Government has declared that “Canada is facing an opioid crisis … the growing number of overdoses and deaths caused by opioids … is a public health emergency” [2]. Furthermore, the problem appears to be worsening: in 2018, there were 4588 opioid-related deaths across Canada, representing a 152% increase compared to the 3023 deaths in 2016 [2]. Opioids, though effective in specific scenarios, are high-risk medications for addiction, tolerance, withdrawal, and fatal overdose [3].

Opioids continue to be the analgesic of choice for postoperative pain control both in the inpatient and outpatient settings across surgical specialties [4]. There remains significant variability in the number of opioids prescribed for common procedures, and the average amount of opioids prescribed per patient has increased over time [5, 6]. Sabatino et al. described the wide variability in prescribing patterns among common orthopaedic procedures [6]. After arthroscopic rotator cuff repair, patients were prescribed, on average, 600 oral morphine equivalents (OMEs) (range 135 to 750, 6]. Furthermore, the average postoperative opioid prescription has also increased from 240 mg OMEs in 2010 to 403 mg in 2016 across common surgical procedures [7].

There is an alarming rate of chronic opioid use following both minor and major surgical procedures [8]. In a large cohort study of Canadians undergoing major surgery, Clarke et al. showed that 3% of opioid naïve patients continued to use opioids over 90 days after surgery [9]. Similarly, Brummett et al. demonstrated that approximately 6% of adult Americans without previous opioid use who underwent various surgical procedures developed chronic opioid use postoperatively [8]. This translates into more than 2 million patients that may transition to chronic opioid use following elective surgery in North America each year.

Orthopaedic surgeons prescribe more opioids than any other surgical specialty [10]. A recent database study across various surgical specialties found that 94% of patients undergoing elective surgery received opioid prescriptions at discharge, and that all orthopaedic surgery patients were well in excess of the recommended opioid prescription guidelines [11]. Despite their widespread use, there are no evidence-based clinical practice guidelines for postoperative prescriptions. Consequently, there is marked variability in prescribing patterns, with the majority of surgeons prescribing an opioid postoperatively [4]. Within orthopaedics, knee and shoulder arthroscopy are the most commonly performed procedures [12]. The literature suggests that surgeons are prescribing anywhere between 90 and 450 OMEs after knee arthroscopy [13]. A recent observational study demonstrated that patients utilized minimal opioids after arthroscopic knee surgery, and that 88% of patients had leftover opioids [14]. These findings are replicated in the shoulder arthroscopy data with the majority of patients receiving excessive opioids postoperatively [15].

In Canada, it is routine practice to prescribe narcotic medications after arthroscopic surgery. Based on our survey of the Arthroscopy Association of Canada, surgeons reported that they prescribe, on average, 156 mg of oral morphine equivalents (OMEs) to patients [16]. This is 2 to 5 times as many OMEs as the median amount that patients actually use after knee arthroscopy (35–86 OMEs) [4, 14]. Only 66% of surgeons discussed the risks of opioids with their patients. On average, these surgeons estimated that only about 12% of their patients requested more opioid medications than initially prescribed. 92% of respondents felt that opioid over-prescription was an issue in surgery as a whole, and 82% believed it was an issue in arthroscopy specifically [16].

### Study objectives

The primary objective of the The Non-Opioid Prescriptions after Arthroscopic Surgery in Canada (NO PAin) trial is to determine, in adult patients aged 18 years and older undergoing outpatient knee or shoulder arthroscopy, whether a non-opioid analgesia approach to postoperative pain, compared to usual care, reduces oral morphine equivalents (OMEs) consumed up to 6 weeks postoperatively. The secondary research objectives are to determine, in this population, the effect of a non-opioid analgesia approach to postoperative pain, compared to usual care on patient-reported pain and satisfaction, quantity of OMEs prescribed, number of opioid refills, and any adverse events up to 6 weeks postoperatively.

## Methods

### Study design

This is a randomized controlled trial (RCT) of 200 patients aged 18 years or older undergoing outpatient knee or shoulder arthroscopy. Patients will be evaluated clinically at 2 and 6 weeks postoperatively. Patients will be recruited from experienced arthroscopic surgeons at 3 hospital sites in Hamilton, Ontario: McMaster University Medical Centre (MUMC), St. Joseph’s Healthcare (SJH), and the Hamilton General Hospital (HGH). Ethics approval for this study was granted by the Hamilton Integrated Research Ethics Board (Version 1.0, 14-January-2021, HIREB #12–670). All research will be conducted according to international standards of Good Clinical Practice and institutional research policies and procedures. Outcome assessors and data analysts will be blinded to the patient allocation. This protocol adheres to the *Standard Protocol Items: Recommendations for Interventional Trials (SPIRIT)* guidelines for reporting of clinical trial protocols (Appendix 1, 2) [17].

### Participant selection

#### Eligibility criteria

The inclusion criteria are patients who are: 1) undergoing outpatient knee or shoulder arthroscopy (Table 1), 2) age 18 or older, 3) have the ability to speak, understand, and read English, and 4) provide informed consent.

**Table 1:** Included procedures

Knee	Shoulder	Shoulder and Knee
ACL reconstruction (with or without LET) MPFL reconstruction (**not** including TTO) Chondroplasty Meniscectomy Meniscal repair Meniscal transplant Microfracture ACI Fixation of unstable osteochondral lesion	Subacromial decompression Rotator cuff repair Shoulder stabilization Superior capsule reconstruction Biceps tenotomy/tenodesis Capsular release SLAP repair	Diagnostic arthroscopy Irrigation and/or debridement Loose body removal Synovectomy

*ACL* anterior cruciate ligament, *LET* lateral extra-articular tenodesis, *MPFL* medial patellofemoral ligament, *TTO* tibial tubercle osteotomy, *ACI* autologous chondrocyte implantation, superior labrum anterior and posterior.

The exclusion criteria include patients who: 1) are taking a home dose of an opioid medication, 2) are involved in ongoing litigation or compensation claims for any injury, 3) are involved in another research study that requires a specific post-operative pain control medication regimen, 4) are undergoing a knee or shoulder arthroscopy procedure that will likely have an operative time greater than 3 h, 5) are undergoing concomitant open surgery, 6) require overnight admission, 7) have a contraindication or allergy to NSAIDs, acetaminophen, or morphine and hydromorphone, 8) are diagnosed with renal disease or cardiac disease, 9) are scheduled for/plan to have an additional surgical procedure during the 6-week follow-up period, and 10) will likely have problems with maintaining follow-up.

#### Participant recruitment and screening

Patients between the ages 18 years or older who are scheduled for a knee or shoulder arthroscopic procedure will be screened prior to their surgery. To screen patients for eligibility, designated study personnel at each clinical site will be in close contact with the participating site investigators (surgeons) and their administrative staff to help identify potential participants. Someone in the patient’s circle of care will ask the potentially eligible patient if they are comfortable being approached about a clinical research study either during a preoperative clinic visit, via a phone call, or email. If the patient agrees, study personnel will contact the patient either in person or via phone call at some point prior to surgery. For all remote consent calls, the study personnel will email the patient the consent form at the beginning of, or prior to the call (Appendix 3). The patient will be informed that they are able to abstain from deciding until the date of the procedure. The study personnel will screen the patient for eligibility by going through all items listed on the Screening Form and if eligible, proceed with obtaining informed consent from the patient. For a remote consent call, patients will be instructed to send a signed scanned copy to the study personnel via email or will be provided a paper consent form to complete prior to their surgery.

#### Randomization

Eligible patients will be randomized by study personnel using the centralized 24-h computerized randomization system (REDCap™ Cloud) that allows for automated internet-based randomization to allocate patients to the control (standard of care) or intervention (non-opioid prescription and infographic) group. Patients will be randomized as close as possible to the time of surgery as permitted by site-specific operating room scheduling.

### Study interventions

#### Non-opioid prescription group

The intervention group was developed in collaboration with surgeons, nurses, and physicians with expertise in peri-operative pain control and will consist of three components:

1. A standardized non-opioid prescription: A prescription for Naproxen 500 mg PO BID PRN × 60 tabs, Acetaminophen 1000 mg PO Q6H PRN × 100,500 mg tabs and Pantoprazole 40 mg PO daily × 30 tabs will be provided to patients in the interventions group. The inclusion of over-the-counter analgesic medications on the prescription accomplishes two goals: a) it legitimizes these medications and suggests that the healthcare providers truly believe in and recommend their use, and b) it allows for patients on Ontario Disability Benefit or Ontario Works (programs to provide assistance with medication costs for eligible groups) access to medications that may otherwise be cost-prohibitive.
2. A limited opioid “rescue prescription”: A prescription of Hydromorphone 1 mg PO Q4H PRN × 10 tabs will be included on a separate prescription. Patients will be instructed to use the opioid prescription only in cases where they are unable to achieve adequate pain control using the non-opioid prescription.
3. Patient education infographic: The infographic will contain information on how to take the prescribed medications, the risks of opioids, and prevalence of opioid misuse and abuse (Appendix 4).

This protocol was developed based on the World Health Organization “analgesic ladder” for pain management and from non-randomized studies of patients undergoing arthroscopic knee and shoulder surgery [18–20]. If the pain is not controlled with the modalities included in the intervention group, patients will be instructed to contact their orthopaedic surgeon for alternative analgesics.

#### Control group

The control group is the current standard of care, which typically consists of a prescription for an opioid [16]. Prior to starting the study, each surgeon investigator will provide the study team with their standard of care prescriptions for each procedure they perform as part of their practice (as per the procedures listed in Table 1). The non-opioid intervention prescription and infographic will also be prepared prior to starting the study. This way, the allocated prescription can be placed on the patient’s chart prior to surgery by study personnel to avoid the potential for surgeon error. The surgeon/resident will review and sign the prescription before it is given to the patient.

In addition to reducing the potential for prescription error, these methods eliminate the risk of surgeons modifying their practice to prescribe less opioids part way through the study if they feel the intervention group is effective as surgeons will be unable to be blinded to the patient’s allocation group.

#### Standardization of Peri-operative pain management

All patients will receive a standardized peri-operative pain management protocol, which will include: a) acetaminophen 1000 mg PO q6h PRN, b) ketorolac (15-30 mg IV × 1), c) ondansetron (4-8 mg PO/IV q8h PRN), d) gravol (25-50 mg PO/IV q6h PRN), e) an extra-articular injection of 10 mL of 0.5% bupivacaine with epinephrine into the soft tissues surrounding the portal sites, f) Oxyneo 10 mg PO × 1 in recovery or 5 mg Oxycodone regular release, and g) hydromorphone 1 mg PO q4h PRN (or Morphine or Oxycodone if intolerant/allergic, see 3.4.1 for details). Note that dose ranges are provided to allow for adjustments based on patient weight if necessary.

### Study outcomes

#### Primary outcome

The primary outcome is the number of total OMEs consumed at 6 weeks postoperatively, as determined by a patient-reported medication diary (up to 2 weeks) and patient reporting (at 6 weeks).

#### Secondary outcomes

Secondary outcomes include: 1) patient-reported pain using a visual analogue scale (VAS), 2) patient-reported satisfaction with pain control using one question from the Hospital Consumer Assessment of Healthcare Providers and Systems (HCAHPS) questionnaire, 3) number of OMEs prescribed, 4) number of opioid refills, and 5) any adverse events up to 6 weeks postoperatively.

#### Outcome measures

Patients will be provided with a medication and pain diary to complete daily from the time of surgery to the 2-week follow-up visit. The medication and pain diary will be used to measure the number of OMEs consumed (primary outcome), the number of OMEs prescribed and refills (secondary outcomes), and daily pain VAS scores (secondary outcome). At the 6-week follow-up patients will also be asked the total amount of opioid medication they have taken.

Patients will complete self or interviewer-administered outcome questionnaires during the routine follow-up visits at 2 weeks and 6 weeks. The VAS will include a 100 mm line, on which patients will be asked to rate their average pain since their surgery. Higher scores indicate higher levels of pain. The VAS is one of the most frequently used pain rating scales in clinical practice and research [21]. The VAS is a validated unidimensional scale that is easy to use, requires no verbal or reading skills, and is sufficiently versatile to be employed in a variety of settings [22–24]. The HCAHPS is a validated and nationally standardized survey designed to evaluate patient perspectives of hospital care [25]. As per previous research evaluating patient satisfaction following orthopaedic procedures, we used a modified question from the HCAHPS questionnaire related to satisfaction with pain relief, answered on a Likert scale (never, sometimes, usually, or always): “In the time after surgery, how often was your pain well controlled?” [19, 26]. For a dichotomous analysis, responses of “always” and “usually” will be grouped as satisfied patients, and responses of “sometimes” or “never” will be grouped as unsatisfied patients. Patient satisfaction will be measured at the 2- and 6-weeks follow-up appointments. Adverse events, defined as any symptom, sign, illness, or experience that develops or worsens in severity during the course of this study, will also be documented (Table 2).

**Table 2:** Schedule of Events

Data Collection	Enrollment	2 Weeks	6 Weeks
Screening and Informed Consent	●		
Enrolment Data (Demographics)	●		
Follow-Up Form		●	●
Medication Diary OMEs consumed OMEs prescribed Opioid refills		#	
Total OMEs consumed			●
Visual Analogue Scale (VAS)		#	●
Patient Satisfaction (Question from HCAHPS)		●	●
Adverse Events		x	x

*X - if applicable, # - daily up to the 2-week visit.*

### Study follow-up

Study participants will be followed at 2 weeks (window between 1 and 3 weeks) and 6 weeks (window between 5 and 7 weeks) postoperatively. Visits that occur outside of these windows must be marked as early or late as appropriate. This follow-up schedule is in accordance with the current practice at each clinical site and does not require extra visits or costs to the patients. Patients who are unable to attend the follow-up appointments will be contacted by telephone to complete the applicable questionnaires and case report forms (CRFs) for all visits up to and including 6 weeks.

### Protecting against sources of Bias

Given that patients in the intervention group will receive a pamphlet explaining how to use their prescription allocation, patient blinding is not feasible. Surgeons cannot be blinded as they will need to sign the prescriptions and provide any necessary advice about the medications being prescribed. However, as described previously, surgeons will provide their standard treatment prior to enrolment to reduce risk of bias. Outcome assessors and data analysts will be blinded.

An independent, blinded Adjudication Committee will be utilized to evaluate all adverse events throughout the duration of the study. The committee is comprised of three orthopaedic surgeons not otherwise involved in the study. All adverse events deemed possibly, probably, or definitely related to the surgery or post-operative pain medication regime will be reviewed. Any disagreements between the Adjudication Committee will be resolved through consensus during regularly scheduled conference calls. If a consensus cannot be reached, additional information will be requested from the participating site.

### Sample size calculation

Based on prior literature, patients undergoing knee and shoulder arthroscopy can be expected to consume a median of about 100 OMEs post-surgery without intervention [4]. We are prescribing 75 OMEs in the intervention group with a rescue opioid prescription. Patients have been shown to consume 25–50% of their prescription depending on whether they are undergoing knee or shoulder arthroscopy, respectively. Given this, we expect that the overall prescription consumption will be 33% of the prescribed amount (i.e. 25 OMEs). Using an alpha-value of 0.05, power of 80%, and a standard deviation of 155 OMEs [4], the required sample size is 68 patients per group, for a total sample size of 136. According to Thoma et al., estimated sample sizes should be increased by 10–40% to allow for loss to follow-up and unforeseen circumstances [27]. Thus, based on the most conservative estimate of this guidance, we will increase our sample size by 40% for a total of 190 patients, rounded to 200 (100 per group). Allowing for patients who need to be excluded, those who choose not to participate, and loss to follow-up, we estimate we will need to screen approximately 300 patients for eligibility for a 66% inclusion rate [28].

### Timeline

**Fig. 1:**
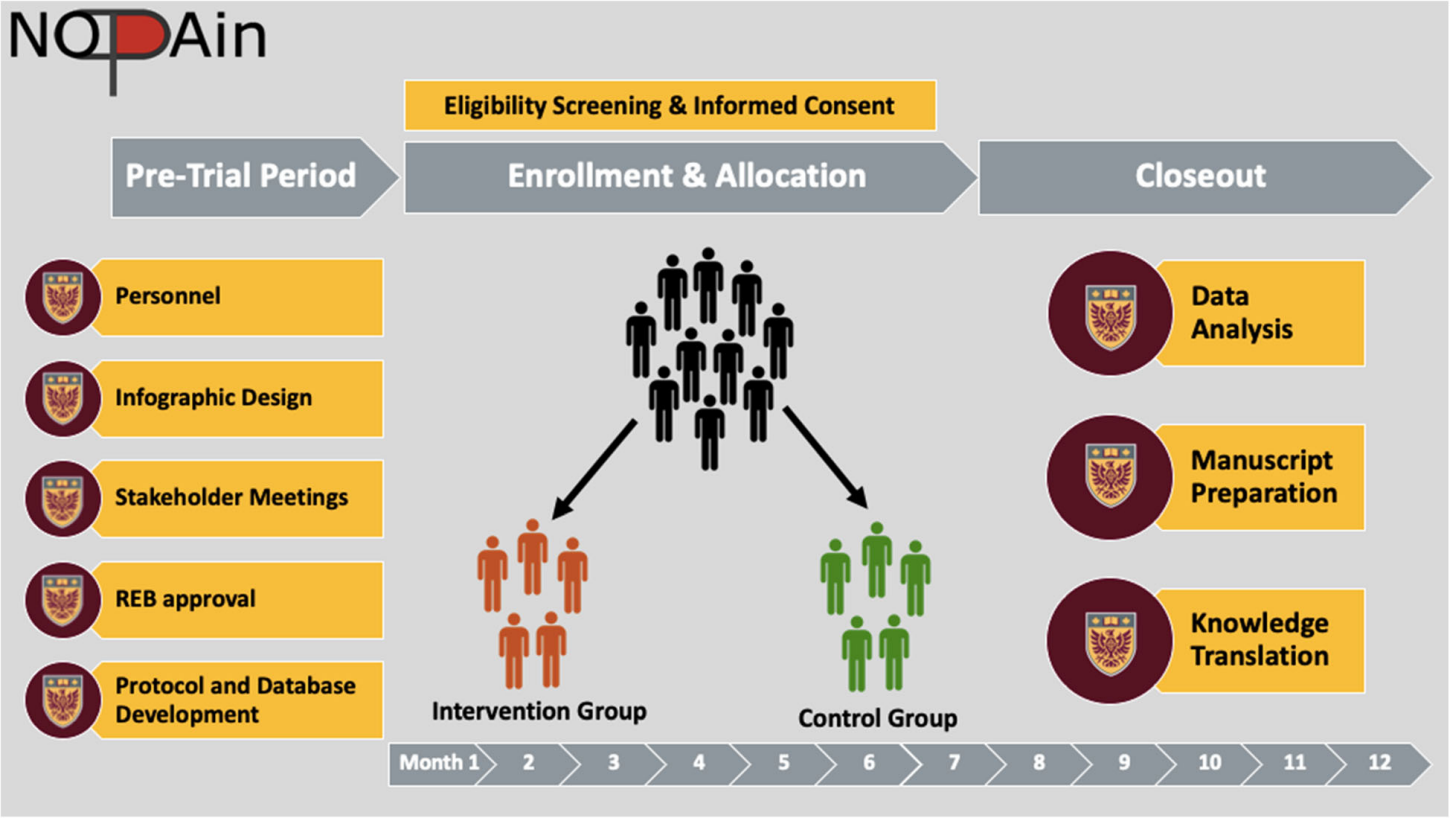
Study Timeline

We estimate we will need to screen approximately 300 patients for eligibility in order to have 200 total included patients. Based on previous caseloads and allowing for holidays, COVID-related delays and other variations, we estimate this will require 8 months for screening and enrolment. We have begun enrolment in March of 2021 and anticipate to complete enrolment by December 2021. Allowing for follow-up completion, data analysis and manuscript preparation, we anticipate the first results for this study will be submitted for peer-review in February 2022 (Fig. 1).

### Statistical plan

We will adopt the intention to treat principle for all analyses—that is, patients will be retained in their randomized groups for all analyses. The baseline characteristics of the patients will be summarized by group, reported as a mean (standard deviation [SD]) or median (first quartile, third quartile) for continuous variables and count (percent) for categorical variables. We will use multiple imputation to handle missing data [28]. No interim analyses are planned. All tests will be 2-sided with α = 0.01. We will use SAS 9.4 (Cary, NC) to perform all analyses.

#### Primary analysis

The number of OMEs consumed will be compared between groups using an independent samples t-test and presented with a *p*-value as well as a mean difference (MD) with 95% confidence intervals (CIs).

#### Secondary analysis

We will perform an independent samples t-tests to test for differences in 2-week VAS scores and OMEs prescribed between groups. We will also plot mean daily VAS scores as per the medication and pain diary over time up to 2 weeks as a descriptive analysis. The proportion of adverse events, satisfied patients, and opioid refills will be compared between groups using an odds ratio. Each secondary outcome will be quantified using descriptive statistics and 95% CIs.

#### Subgroup/sensitivity analyses

We plan to conduct 3 subgroup analyses comparing 1) shoulder versus knee arthroscopy patients; 2) patients who received a regional block of any kind as a part of their anesthetic versus those who did not; and 3) males versus females. We plan to perform a linear regression and include treatment by subgroup interactions to assess whether the magnitude of the treatment effect is significantly different between these subgroups.

### Data management

The CRFs will be the primary data collection tool for the study. All data requested on the CRF must be recorded. An Electronic Data Capture (EDC) system (REDCap™ Cloud) will be used to submit data to the Methods Centre located at McMaster University. Site personnel will receive a unique login and password for the REDCap Cloud system and will be able to view and modify data for participants recruited at their clinical site. Upon receipt of the data, the personnel at the Methods Centre will make a visual check of the data and they will query all missing data, implausible data, and inconsistencies.

### Monitoring

#### Safety monitoring

An independent Medical Monitor will be sent regular updates to monitor the study data for safety. The Medical Monitor will provide medical expertise for study oversight and safety concerns and is required to provide recommendations about starting, continuing, and stopping the study.

#### Interim analysis

No interim analyses are planned, and the study will not be stopped early for benefit. The Medical Monitor will review frequent safety reports and will make judgments on the strength of evidence and the absolute magnitude and seriousness of any safety signals. The Medical Monitor may make recommendations to the Principal Investigators to stop the study due to harm.

#### Data Monitoring

The REDCap Cloud EDC system will be programmed with a number of edit and logic checks that are automatically triggered during data entry by study personnel and during data validation by Methods Centre personnel. While validating data, Methods Centre personnel will review each automatically generated query and create queries to clinical sites as appropriate.

### Ethical considerations

#### Consent

Any patients who are deemed to meet all eligibility criteria will be approached to discuss participation in the trial by a member of the study team who is knowledgeable about the study. Consent may be obtained electronically or using pen and paper consent forms, as approved by the local ethics board. If potential participants are contacted by telephone, documenting written informed consent will occur. The process of obtaining and documenting informed consent will be completed in accordance with local Good Clinical Practice recommendations and HiREB requirements.

#### Confidentiality

Information about study participants will be kept confidential and will be managed in accordance with the following rules: 1) all study-related information will be stored securely at the clinical site, 2) all study participant information will be stored in locked file cabinets and be accessible only to study personnel, 3) all CRFs will be identified only by a unique coded participant number, and 4) all records that contain participant names, or other identifying information (e.g. consent forms and contact information forms), will be stored separately from the study records that are identified only by the coded participant number and initials.

## Discussion

The rationale for the NO PAin trial includes: 1) the ongoing opioid epidemic and overprescribing in orthopaedics; 2) a focus on a set of procedures in which pain management is likely to be successful with minimal opioids, based on prior evidence and the minimally invasive nature of the surgery; 3) the demonstration in other surgical specialties of feasible non-opioid protocols for pain management; and 4) a lack of evidence upon which to develop post-operative pain management guidelines for patients undergoing arthroscopic surgery.

The NO PAin trial will be among the first within orthopaedic surgery, and specifically arthroscopic surgery. Importantly, it is a recognition of the fact that as more orthopaedic surgery patients are discharged home on the day of their surgery, or shortly thereafter, there needs to be a greater focus on patients’ analgesic protocols after discharge [29]. The majority of opioid reduction studies in orthopaedic surgery and arthroscopy are focused on the immediate peri-operative time period, and the majority of interventions occur while patients are in hospital [30]. While such measures represent an important way to minimize patient pain and opioid usage while in hospital, the impact on post-hospital pain and opioid utilization is less clear.

The utilization of a multifaceted education and pain management strategy also recognizes the importance of patient and healthcare worker education as a component of a successful opioid reduction strategy. In our experience, most patients are unaware of which non-opioid medications they can take while on opioids, and how different classes of medications interact. This can potentially lead to avoidance of non-opioid analgesics, and thus lead patients to consume more opioid medications than they may need. Furthermore, previous literature has shown that most patients never receive information about how to dispose of excess opioids, and most do not already know how to do this [31]. Thus, this multifaceted approach, which has been shown to be effective in laparoscopic general surgery, holds promise for addressing multiple components of opioid over-prescription and overuse [32].

A prospective, randomized evaluation of this relatively inexpensive intervention will demonstrate whether an explicit effort to reduce the number of opioids prescribed actually results in a reduction in the amount of opioids consumed and help to inform future studies and guidelines.

## Supplementary Information

**Additional file 1.** SPIRIT Checklist.

**Additional file 2.** Study Developed Questionnaire.

**Additional file 3.** Consent Form.

**Additional file 4.** Infographic.

## Data Availability

Not applicable.

## Abbreviations

NO PAin: Non-Opioid Prescriptions after Arthroscopic Surgery in Canada
RCT: Randomized controlled trial
OME: Oral morphine equivalent
HIREB: Hamilton Integrated Research Ethics Board
SD: Standard Deviation
CI: Confidence interval
ACL: Anterior cruciate ligament
LET: Lateral extra-articular tenodesis
MPFL: Medial patellofemoral ligament
TTO: Tibial tubercle osteotomy
ACI: Autologous chondrocyte implantation
VAS: Visual analogue scale
HCAPHPS: Hospital Consumer Assessment of Healthcare Providers and Systems
CRF: Case report form

## References

[1] Narcotic Drugs Technical Report 2016. https://www.incb.org/incb/en/narcotic-drugs/Technical_Reports/2016/narcotic-drugs-technical-report-2016.html. Accessed 14 Oct 2020.

[2] Responding to Canada’s opioid crisis - Canada.ca. https://www.canada.ca/en/health-canada/services/substance-use/problematic-prescription-drug-use/opioids/responding-canada-opioid-crisis.html. Accessed 14 Oct 2020.

[3] Kosten TR, George TP (2002). The Neurobiology of Opioid Dependence: Implications for Treatment. Sci Pract Perspect.

[4] Fujii MH, Hodges AC, Russell RL, Roensch K, Beynnon B, Ahern TP (2018). Post-Discharge Opioid Prescribing and Use after Common Surgical Procedure. J Am Coll Surg.

[5] Hill MV, McMahon ML, Stucke RS, Barth RJ (2017). Wide Variation and Excessive Dosage of Opioid Prescriptions for Common General Surgical Procedures. Ann Surg.

[6] Sabatino MJ, Kunkel ST, Ramkumar DB, Keeney BJ, Jevsevar DS (2018). Excess Opioid Medication and Variation in Prescribing Patterns Following Common Orthopaedic Procedures. J Bone Joint Surg Am.

[7] Larach DB, Waljee JF, Hu H-M, Lee JS, Nalliah R, Englesbe MJ (2020). Patterns of Initial Opioid Prescribing to Opioid-Naive Patients. Ann Surg.

[8] Brummett CM, Waljee JF, Goesling J, Moser S, Lin P, Englesbe MJ (2017). New Persistent Opioid Use After Minor and Major Surgical Procedures in US Adults. JAMA Surg.

[9] Rates and risk factors for prolonged opioid use after major surgery: population based cohort study. BMJ. https://www.bmj.com/content/348/bmj.g1251. Accessed 14 Oct 2020. 10.1136/bmj.g1251 PMC3921439 24519537

[10] Volkow ND, McLellan TA, Cotto JH, Karithanom M, Weiss SRB (2011). Characteristics of Opioid Prescriptions in 2009. JAMA..

[11] Thiels CA, Anderson SS, Ubl DS, Hanson KT, Bergquist WJ, Gray RJ (2017). Wide Variation and Overprescription of Opioids After Elective Surgery. Ann Surg.

[12] Garrett WE, Swiontkowski MF, Weinstein JN, Callaghan J, Rosier RN, Berry DJ (2006). American Board of Orthopaedic Surgery Practice of the Orthopaedic Surgeon: Part-II, certification examination case mix. J Bone Joint Surg Am.

[13] Gardner V, Gazzaniga D, Shepard M, Grumet R, Rubin B, Dempewolf M (2018). Monitoring Postoperative Opioid Use Following Simple Arthroscopic Meniscectomy: A Performance-Improvement Strategy for Prescribing Recommendations and Community Safety. JB JS Open Access.

[14] Wojahn RD, Bogunovic L, Brophy RH, Wright RW, Matava MJ, Green JRI (2018). Opioid Consumption After Knee Arthroscopy. JBJS..

[15] Kumar K, Gulotta LV, Dines JS, Allen AA, Cheng J, Fields KG (2017). Unused Opioid Pills After Outpatient Shoulder Surgeries Given Current Perioperative Prescribing Habits. Am J Sports Med.

[16] Ekhtiari S, Horner NS, Shanmugaraj A, Duong A, Simunovic N, Ayeni OR. Narcotic Prescriptions following Knee and Shoulder Arthroscopy: A Survey of the Arthroscopy Association of Canada. Cureus. 2020;12. 10.7759/cureus.7856. 10.7759/cureus.7856 PMC7255063 32483506

[17] Chan A-W, Tetzlaff JM, Altman DG, Laupacis A, Gøtzsche PC, Krleža-Jerić K (2013). SPIRIT 2013 statement: defining standard protocol items for clinical trials. Ann Intern Med.

[18] Anekar AA, Cascella M (2020). WHO Analgesic ladder. StatPearls [Internet].

[19] Daniels SD, Garvey KD, Collins JE, Matzkin EG (2019). Patient Satisfaction With Nonopioid Pain Management Following Arthroscopic Partial Meniscectomy and/or Chondroplasty. Arthroscopy..

[20] Moutzouros V, Jildeh TR, Khalil LS, Schwartz K, Hasan L, Matar RN (2020). A multimodal protocol to diminish pain following common orthopedic sports procedures: Can we eliminate postoperative opioids?. Arthroscopy.

[21] Price DD, McGrath PA, Rafii A, Buckingham B (1983). The validation of visual analogue scales as ratio scale measures for chronic and experimental pain. Pain..

[22] Jensen M, Karoly P, Braver S (1986). The measurement of clinical pain intensity: A comparison of six methods. Pain..

[23] Collins SL, Moore RA, McQuay HJ (1997). The visual analogue pain intensity scale: what is moderate pain in millimetres?. PAIN..

[24] Ho K, Spence J, Murphy MF (1996). Review of Pain-Measurement Tools. Ann Emerg Med.

[25] HCAHPS: Patients’ Perspectives of Care Survey | CMS. https://www.cms.gov/Medicare/Quality-Initiatives-Patient-Assessment-Instruments/HospitalQualityInits/HospitalHCAHPS. Accessed 13 Nov 2020.

[26] Bot AGJ, Bekkers S, Arnstein PM, Smith RM, Ring D (2014). Opioid Use After Fracture Surgery Correlates With Pain Intensity and Satisfaction With Pain Relief. Clin Orthop Relat Res.

[27] Thoma A, Farrokhyar F, McKnight L, Bhandari M (2010). How to optimize patient recruitment. Can J Surg.

[28] Statistical analysis with missing data | Guide books. https://dl.acm.org/doi/book/10.5555/21412. Accessed 13 Nov 2020.

[29] Kaye AD, Urman RD, Cornett EM, Hart BM, Chami A, Gayle JA (2019). Enhanced recovery pathways in orthopedic surgery. J Anaesthesiol Clin Pharmacol.

[30] Gazendam A, Ekhtiari S, Horner NS, Nucci N, Dookie J, Ayeni OR. Perioperative nonopioid analgesia reduces postoperative opioid consumption in knee arthroscopy: a systematic review and meta-analysis. Knee Surg Sports Traumatol Arthrosc. 2020. 10.1007/s00167-020-06256-2. 10.1007/s00167-020-06256-2 32889557

[31] AlAzmi A, AlHamdan H, Abualezz R, Bahadig F, Abonofal N, Osman M. Patients' Knowledge and Attitude toward the Disposal of Medications. J Pharm (Cairo). 2017;2017:8516741. 10.1155/2017/8516741. Epub 2017 Oct 10. 10.1155/2017/8516741 PMC5654249 29130019

[32] Hartford LB, Van Koughnett JAM, Murphy PB, Vogt KN, Hilsden RJ, Clarke CFM (2019). Standardization of Outpatient Procedure (STOP) Narcotics: A Prospective Non-Inferiority Study to Reduce Opioid Use in Outpatient General Surgical Procedures. J Am Coll Surg.

