# Correction to: Protocol for a multicenter randomized controlled trial comparing a non-opioid prescription to the standard of care for pain control following arthroscopic knee and shoulder surgery

**DOI:** 10.1186/s12891-021-04474-4

**Published:** 2021-07-10

**Authors:** The NO PAin Investigators, Aaron Gazendam, Seper Ekhtiari, Nolan S. Horner, Nicole Simunovic, Andrew Duong, Darren de Sa, Devin Peterson, Matthew Denkers, Vickas Khanna, Anthony Adili, Jaydeep Moro, Moin Khan, Olufemi R. Ayeni

**Affiliations:** Department of Surgery, Division of Orthopaedic Surgery, McMaster University, 1200 Main St West, 4E15, Hamilton, ON L8N 3Z5 Canada

**Correction to: BMC Musculoskelet Disord 22, 471 (2021)**

**https://doi.org/10.1186/s12891-021-04354-x**

Following the publication of the original article [1] the authors noticed that the authors “Aaron Gazendam, Seper Ekhtiari, Nolan S. Horner, Nicole Simunovic, Andrew Duong, Darren de Sa, Devin Peterson, Matthew Denkers, Vickas Khanna, Anthony Adili, Jaydeep Moro, Moin Khan, Olufemi R. Ayeni”are still not listed as the NO PAIN investigators in the published version. This was instructed during proofing stage but it was not implemented.

The original article [1] has been updated.
