# Supplementary material for: Protocol for a multicenter randomized controlled trial comparing a non-opioid prescription to the standard of care for pain control following arthroscopic knee and shoulder surgery
Source: BMC Musculoskelet Disord. 2021 May 22;22:471. doi: 10.1186/s12891-021-04354-x (PMC8141233; doi:10.1186/s12891-021-04354-x)
Supplement: Supplementary file 2 — Additional file 2. Study Developed Questionnaire. [file 12891_2021_4354_MOESM2_ESM.pdf]

Patient Study ID Number: ☐☐ ☐☐☐☐ NO Pain

PATIENT MEDICATION AND PAIN DIARY

INSTRUCTIONS: Please complete this diary every day until your 2-week follow-up visit. Try to be as thorough as possible. If you run out of room for a given day, please write on the back of the page. **Please remember to bring the pages you filled out to your 2-week follow-up visit with the surgeon.**

Date	Medication Name	Dose of 1 Pill (mg)	Number of Pills Taken	Comments (Side effects?)
☐☐ ☐☐ 2 0 ☐☐<br>DD MM YYYY				
Please rate your pain today:	No pain — Moderate pain — Unbearable pain			

Patient Study ID Number: ☐☐ ☐☐☐☐

NO Pain

PATIENT MEDICATION AND PAIN DIARY

INSTRUCTIONS: Please complete this diary every day until your 2-week follow-up visit. Try to be as thorough as possible. If you run out of room for a given day, please write on the back of the page. **Please remember to bring the pages you filled out to your 2-week follow-up visit with the surgeon.**

Date	Medication Name	Dose of 1 Pill (mg)	Number of Pills Taken	Comments (Side effects?)
☐☐ ☐☐ 2 0 ☐☐<br>DD MM YYYY				
Please rate your pain today:	No pain ... Moderate pain ... Unbearable pain			

Patient Study ID Number: ☐☐ ☐☐☐☐ NO Pain

PATIENT MEDICATION AND PAIN DIARY

INSTRUCTIONS: Please complete this diary every day until your 2-week follow-up visit. Try to be as thorough as possible. If you run out of room for a given day, please write on the back of the page. **Please remember to bring the pages you filled out to your 2-week follow-up visit with the surgeon.**

Date	Medication Name	Dose of 1 Pill (mg)	Number of Pills Taken	Comments (Side effects?)
☐☐ ☐☐ 2 0 ☐☐<br>DD MM YYYY				
Please rate your pain today:	No pain	Moderate pain		Unbearable pain

Patient Study ID Number: ☐☐ ☐☐☐☐ NO Pain

PATIENT MEDICATION AND PAIN DIARY

INSTRUCTIONS: Please complete this diary every day until your 2-week follow-up visit. Try to be as thorough as possible. If you run out of room for a given day, please write on the back of the page. **Please remember to bring the pages you filled out to your 2-week follow-up visit with the surgeon.**

Date	Medication Name	Dose of 1 Pill (mg)	Number of Pills Taken	Comments (Side effects?)
☐☐ ☐☐ 2 0 ☐☐<br>DD MM YYYY				
Please rate your pain today:	No pain — Moderate pain — Unbearable pain			

Patient Study ID Number: ☐☐ ☐☐☐☐ NO Pain

PATIENT MEDICATION AND PAIN DIARY

INSTRUCTIONS: Please complete this diary every day until your 2-week follow-up visit. Try to be as thorough as possible. If you run out of room for a given day, please write on the back of the page. **Please remember to bring the pages you filled out to your 2-week follow-up visit with the surgeon.**

Date	Medication Name	Dose of 1 Pill (mg)	Number of Pills Taken	Comments (Side effects?)
☐☐ ☐☐ 2 0 ☐☐<br>DD MM YYYY				
Please rate your pain today:	No pain	Moderate pain	Unbearable pain	

Patient Study ID Number: ☐☐ ☐☐☐☐ NO Pain

PATIENT MEDICATION AND PAIN DIARY

INSTRUCTIONS: Please complete this diary every day until your 2-week follow-up visit. Try to be as thorough as possible. If you run out of room for a given day, please write on the back of the page. **Please remember to bring the pages you filled out to your 2-week follow-up visit with the surgeon.**

Date	Medication Name	Dose of 1 Pill (mg)	Number of Pills Taken	Comments (Side effects?)
☐☐ ☐☐ 2 0 ☐☐<br>DD MM YYYY				
Please rate your pain today:	No pain — Moderate pain — Unbearable pain			

Patient Study ID Number: ☐☐ ☐☐☐☐

NO Pain

PATIENT MEDICATION AND PAIN DIARY

INSTRUCTIONS: Please complete this diary every day until your 2-week follow-up visit. Try to be as thorough as possible. If you run out of room for a given day, please write on the back of the page. **Please remember to bring the pages you filled out to your 2-week follow-up visit with the surgeon.**

Date	Medication Name	Dose of 1 Pill (mg)	Number of Pills Taken	Comments (Side effects?)
☐☐ ☐☐ 2 0 ☐☐<br>DD MM YYYY				
Please rate your pain today:	No pain — Moderate pain — Unbearable pain			

Patient Study ID Number: ☐☐ ☐☐☐☐ NO Pain

PATIENT MEDICATION AND PAIN DIARY

INSTRUCTIONS: Please complete this diary every day until your 2-week follow-up visit. Try to be as thorough as possible. If you run out of room for a given day, please write on the back of the page. **Please remember to bring the pages you filled out to your 2-week follow-up visit with the surgeon.**

Date	Medication Name	Dose of 1 Pill (mg)	Number of Pills Taken	Comments (Side effects?)
☐☐ ☐☐ 2 0 ☐☐<br>DD MM YYYY				
Please rate your pain today:	No pain — Moderate pain — Unbearable pain			

Patient Study ID Number: ☐☐ ☐☐☐☐

NO Pain

PATIENT MEDICATION AND PAIN DIARY

INSTRUCTIONS: Please complete this diary every day until your 2-week follow-up visit. Try to be as thorough as possible. If you run out of room for a given day, please write on the back of the page. **Please remember to bring the pages you filled out to your 2-week follow-up visit with the surgeon.**

Date	Medication Name	Dose of 1 Pill (mg)	Number of Pills Taken	Comments (Side effects?)
☐☐ ☐☐ 2 0 ☐☐<br>DD MM YYYY				
Please rate your pain today:	No pain — Moderate pain — Unbearable pain			

11-Nov-2020

Version 2.0

Patient Study ID Number: ☐☐ ☐☐☐☐

NO Pain

PATIENT MEDICATION AND PAIN DIARY

INSTRUCTIONS: Please complete this diary every day until your 2-week follow-up visit. Try to be as thorough as possible. If you run out of room for a given day, please write on the back of the page. **Please remember to bring the pages you filled out to your 2-week follow-up visit with the surgeon.**

Date	Medication Name	Dose of 1 Pill (mg)	Number of Pills Taken	Comments (Side effects?)
☐☐ ☐☐ 2 0 ☐☐<br>DD MM YYYY				
Please rate your pain today:	No pain — Moderate pain — Unbearable pain			

Patient Study ID Number: ☐☐ ☐☐☐☐ NO Pain

PATIENT MEDICATION AND PAIN DIARY

INSTRUCTIONS: Please complete this diary every day until your 2-week follow-up visit. Try to be as thorough as possible. If you run out of room for a given day, please write on the back of the page. **Please remember to bring the pages you filled out to your 2-week follow-up visit with the surgeon.**

Date	Medication Name	Dose of 1 Pill (mg)	Number of Pills Taken	Comments (Side effects?)
☐☐ ☐☐ 2 0 ☐☐<br>DD MM YYYY				
Please rate your pain today:	No pain		Moderate pain	Unbearable pain

Patient Study ID Number: __ __ __ __ __ __ NO Pain

PATIENT MEDICATION AND PAIN DIARY

INSTRUCTIONS: Please complete this diary every day until your 2-week follow-up visit. Try to be as thorough as possible. If you run out of room for a given day, please write on the back of the page. **Please remember to bring the pages you filled out to your 2-week follow-up visit with the surgeon.**

Date	Medication Name	Dose of 1 Pill (mg)	Number of Pills Taken	Comments (Side effects?)
__ __ / __ __ / 2 0 __ __<br>DD MM YYYY				
Please rate your pain today:	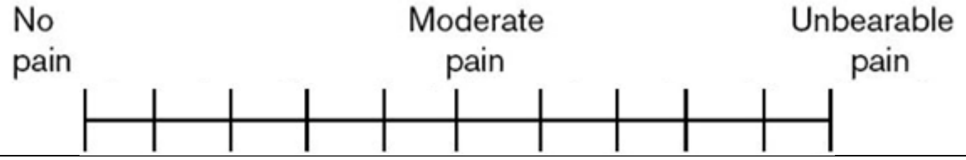 			

Patient Study ID Number: ☐☐ ☐☐☐☐

NO Pain

PATIENT MEDICATION AND PAIN DIARY

INSTRUCTIONS: Please complete this diary every day until your 2-week follow-up visit. Try to be as thorough as possible. If you run out of room for a given day, please write on the back of the page. **Please remember to bring the pages you filled out to your 2-week follow-up visit with the surgeon.**

Date	Medication Name	Dose of 1 Pill (mg)	Number of Pills Taken	Comments (Side effects?)
☐☐ ☐☐ 2 0 ☐☐<br>DD MM YYYY				
Please rate your pain today:	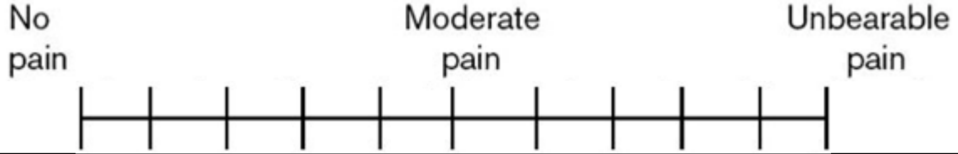			

Patient Study ID Number: ☐☐ ☐☐☐☐ NO Pain

PATIENT MEDICATION AND PAIN DIARY

INSTRUCTIONS: Please complete this diary every day until your 2-week follow-up visit. Try to be as thorough as possible. If you run out of room for a given day, please write on the back of the page. **Please remember to bring the pages you filled out to your 2-week follow-up visit with the surgeon.**

Date	Medication Name	Dose of 1 Pill (mg)	Number of Pills Taken	Comments (Side effects?)
☐☐ ☐☐ 2 0 ☐☐<br>DD MM YYYY				
Please rate your pain today:	No pain	Moderate pain	Unbearable pain	
