# Supplementary material for: Protocol for a multicenter randomized controlled trial comparing a non-opioid prescription to the standard of care for pain control following arthroscopic knee and shoulder surgery
Source: BMC Musculoskelet Disord. 2021 May 22;22:471. doi: 10.1186/s12891-021-04354-x (PMC8141233; doi:10.1186/s12891-021-04354-x)
Supplement: Supplementary file 3 — Additional file 3. Consent Form. [file 12891_2021_4354_MOESM3_ESM.pdf]

CONSENT TO PARTICIPATE IN A RESEARCH STUDY (Participant)

Non-**O**pioid **P**rescriptions after **A**rthroscopic Surgery **in** Canada: A Randomized Controlled Trial

Locally Responsible Investigator and Principal Investigator (Supervisor): Dr. Olufemi Ayeni, Department of Surgery, McMaster University Medical Centre, 905-527-4322 ext. 44982

Principal Investigator: Dr. Nolan Horner, MD, Department of Surgery, McMaster University

Co-Principal Investigators: Dr. Aaron Gazendam, MD, Department of Surgery, McMaster University; Dr. Seper Ekhtiari, MD, Department of Surgery, McMaster University

Co-Investigators: Dr. Anthony Adili, Dr. Moin Khan, Dr. Vickas Khanna, Dr. Jaydeep Moro, Department of Surgery, St. Joseph's Healthcare Hamilton; Dr. Darren de SA, Dr. Devin Peterson, Department of Surgery, McMaster University Medical Centre; Dr. Matthew Denkers, Department of Surgery, Hamilton General Hospital

Funding Source: Hamilton Health Sciences

INTRODUCTION

You are being invited to participate in a research study because you have been diagnosed with a knee or shoulder injury requiring arthroscopic surgery to fix.

In order to decide whether or not you want to be part of this research study, you should understand what is involved and the potential risks and benefits. This form gives detailed information about the research study, which will be discussed with you. Once you understand the study, you will be asked to sign this form if you wish to participate. Please take your time to make your decision. Feel free to discuss it with your friends and family, or your family physician.

McMaster University and the investigators are receiving compensation to cover the costs of conducting the study. Your surgeon and all members of his team will not receive any personal payments or other compensation from the Sponsor related to this study.

PURPOSE

Canadians use more opioids than people in any country except for the United States. Orthopaedic surgeons prescribe more opioids (also known as narcotics) than any other type of surgeon. There is currently an opioid crisis in Canada. Opioids have the potential to be highly addictive and can cause serious harm or even death if taken in excess. In Hamilton, the rates of opioid-related deaths are double the provincial rate and are on the rise each year. They are routinely prescribed after surgical operations even though there is little evidence to suggest that they are beneficial or needed. Knee and shoulder arthroscopy (or "scopes")

are among the most common orthopaedic surgeries. In fact, they are the most common orthopaedic surgeries in the world. These surgeries are done through minimally invasive “keyhole” incisions, which are supposed to cause less pain than bigger open surgeries. Even though these types of surgeries help reduce surgical pain, the majority of Canadian surgeons still prescribe opioids after these arthroscopic surgeries. Previous research has shown that non-opioid pain medications, including anti-inflammatories do provide adequate pain relief after similar minimally invasive ‘keyhole” abdominal surgeries.

The purpose of this research project is to determine if we can avoid the prescription of opioid medications after knee and shoulder scopes without having a negative effect on patient pain levels and satisfaction with pain control after arthroscopic surgery. It is not known if opioid pain control medications are necessary for adequate pain control after arthroscopic surgery or if safer medications such as Tylenol (Acetaminophen) or anti-inflammatory medications will provide adequate pain control. Currently there exists no guidelines for surgeons on appropriate pain control prescriptions after arthroscopic surgery. Evidence from research on similar procedures strongly suggests that opioid prescriptions may be unnecessary.

This study is a research study with participants recruited at multiple hospitals in Hamilton. There will be approximately 200 patients in this study from McMaster University Medical Centre, Hamilton General Hospital and St. Joseph’s Hospital. Not all patients receiving shoulder or knee arthroscopy surgery will be eligible to participate in this study due to the inclusion and exclusion criteria. Your participation will last approximately 6 weeks.

EXPLANATION OF STUDY PROCEDURES

If you agree to participate in this study, your treatment will be determined by a process called randomization. Randomization means that your treatment group will be decided by chance like flipping a coin. You will have a 1 in 2 chance of receiving one of the following treatments:

1. Patients will receive prescriptions for non-opioid medication in the form of an oral anti-inflammatory medication (Naproxen) and acetaminophen (Tylenol). In addition to this pain medication, the patients will also be given an educational infographic (pamphlet) in their pre-operative package, which will talk about the risks of opioid medications, and provide information about other methods of pain control after surgery. Additionally, these patients will be provided a **“rescue prescription”** for a small number of opioid pills. This prescription is to only be filled and used if the patients cannot manage their pain with the non-opioid medications provided.
2. Patients in the second group will receive prescriptions for whatever pain medication their surgeon usually prescribes after surgery. The majority of surgeons at Hamilton Health Sciences routinely prescribe an opioid medication following arthroscopic surgery.

None of these treatments are experimental. If you need more pain medications, you can contact either your surgeon or an orthopaedic surgery resident 24/7, who can then fax a script to the closest pharmacy or provide it to you in-person at the hospital

STUDY COMMITMENT

We will follow up with you at 2 and 6 weeks after surgery, which are part of your routine follow-up visits after this type of surgery. We will ask if, and how many opioid pills you took, and if you followed the safe storage and disposal habits described in the infographic. We will also ask how much pain you had on average on a 0-100 scale, and how satisfied you are (also from 0-100) with the post-operative pain

management, as well as if you requested a refill of your medication and any adverse events you may have had.

POSSIBLE RISKS

Any treatment has possible side effects. The surgical procedures used in this study are commonly performed, but there IS a small chance of adverse events, including, but not limited to infection (<1%), stiffness (1-8%) , and/or blood clot (<1%).

After surgery, the use of opioids or non-opioids may cause some side effects. You may experience all, some, or none of the side effects listed. These may include, but not limited to: gastrointestinal upset (10-30%), dermatologic/rash (10-25%) and drowsiness or decreased level of consciousness (10-20%). There is always the risk of very uncommon or previously unknown side effects, but being randomized to either of the treatment strategies will not put you at greater risk for any of these issues.

POSSIBLE BENEFITS

Possible benefits may include a great reduction in pain levels and increase in function after surgery. Both pain management strategies have been developed to maximize patient outcomes and are considered acceptable and within the current standards of care. It is not possible to know whether or not you will benefit from participating in this study. You understand that the information gained from this study may be used scientifically and may be helpful to others.

COMPENSATION

Participants in this study will not be paid or reimbursed for their time for their participation in this study. However, participants that complete study visits in person will be provided with parking vouchers to cover their parking costs for that visit. The information you provide may benefit future patients.

ALTERNATIVE (OTHER) TREATMENTS

You can still receive evaluation and treatment for your condition if you do not participate in this study. Discuss any alternative treatments with your regular doctor and/or the study doctor before you decide to participate in the study. Your decision is entirely up to you. If you decide not to participate in the study, you will not be penalized or lose any benefits and your decision will not affect your relationship with your doctor or hospital. Choosing not to take part in the study will in no way affect your care or treatment.

VOLUNTARY PARTICIPATION

Participation in this study is completely voluntary. You may refuse to answer any questions you do not want to answer and remain in the study. You are free to withdraw at any time without affecting the quality of the care you receive at this institution, and by signing this form you do not waive your legal rights. When you withdraw your permission, no new health information will be gathered after that date. Information that has already been gathered may still be used. If you would like to withdraw from this study, you will need to provide written or verbal confirmation to the study coordinator (Andrew Duong, Department of Surgery, McMaster University, 1200 Main St. W, HSC 1E1, Hamilton, ON L8N 3Z5, 905-923-2126).

NEW INFORMATION

During this study, you will be told of any new information that may affect your decision to participate in this study. A Medical Monitor will be reviewing the data from this research throughout the study. They

will notify your doctor of any new information that you need to be told about. If the Medical Monitor decides to end the study early, you will be notified directly.

AUTHORIZATION TO USE AND DISCLOSE (RELEASE) MEDICAL INFORMATION

Your data will not be shared with anyone except with your consent or as required by law. All personal information such as your name, address, phone number, OHIP number, family physician's name will be removed from the data and will be replaced with a number. A list linking the number with your name will be kept in a secure place, separate from your file. The data, with identifying information removed will be securely stored in a locked office in the research department. This data will be securely stored for 10 years. If the results of the study are published or presented, your name will not be used and no information that discloses your identity will be released or published without your specific consent to the disclosure. If you have any questions about the privacy of your health information, please ask your study doctor.

CONTACT FOR QUESTIONS

For more information concerning this study and research-related risks or injuries, you may contact the Investigators, Drs. Horner, Gazendam or Ekhtiari at no.pain.hamilton@gmail.com

Locally Responsible Investigator and Principal Investigator: Dr. Nolan Horner, Department of Surgery, McMaster University Medical Centre, 1200 Main St. W, HSC 1E1, Hamilton, ON L8N 3Z5, 613-583-1067).

CONSENT TO PARTICIPATE

Participant:
I have read the preceding information thoroughly. I have had an opportunity to ask questions and all of my questions have been answered to my satisfaction. I agree to participate in this study. I understand that I will receive a signed copy of this form.

Name of Participant (please print)	Signature	Date

Person obtaining consent:
I have discussed this study in detail with the participant. I believe the participant understands what is involved in this study.

Name, Role in Study	Signature	Date

INVESTIGATOR STATEMENT
I have carefully explained to the participant the nature and purpose of this study. The participant signing this consent form has (1) been given the time and place to read and review this consent form; (2) been given an opportunity to ask question regarding the nature, risks and benefits of participation in this research study; and (3) appears to understand the nature and purpose of the study and the demands required of participation. The participant has signed this consent form prior to having any study-related procedures performed.

Name of Investigator	Signature	Date

This study has been reviewed by the Hamilton Integrated Research Ethics Board (HiREB). The HiREB is responsible for ensuring that participants are informed of the risks associated with the research, and that participants are free to decide if participation is right for them. If you have any questions about your rights as a research participant, please call the Office of the REB Chair, HiREB at 905.521.2100 x 42013.
