# Supplementary material for: Protocol for a multicenter randomized controlled trial comparing a non-opioid prescription to the standard of care for pain control following arthroscopic knee and shoulder surgery
Source: BMC Musculoskelet Disord. 2021 May 22;22:471. doi: 10.1186/s12891-021-04354-x (PMC8141233; doi:10.1186/s12891-021-04354-x)
Supplement: Supplementary file 4 — Additional file 4. Infographic. [file 12891_2021_4354_MOESM4_ESM.pdf]

Recommendations:

- The use of ice, heat or physical therapy can reduce inflammation and pain.
- Your pain should subside daily after your surgery. Many patients do not use any opioids after the third day after surgery.
- Complete your medication diary, and keep track of your medication use, including opioids and non-opioid medications.
- Ask your doctor about whether you need additional pain management strategies, including prescription renewals or alternative treatments.
- With opioids, there is a fine balance between effective pain control and dangerous side effects. If you have questions, please contact the Research Team.

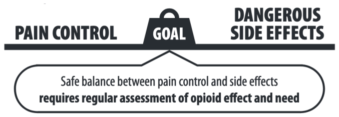

If you have any questions, please contact your Doctor, or the Research Team

Study Contact Information

Research Team

no.pain.hamilton@gmail.com

Research Coordinator

Andrew Duong
duonga@mcmaster.ca
905-923-2126

Non-Opioid Prescriptions after Arthroscopic Surgery in Canada: A Randomized Controlled Trial

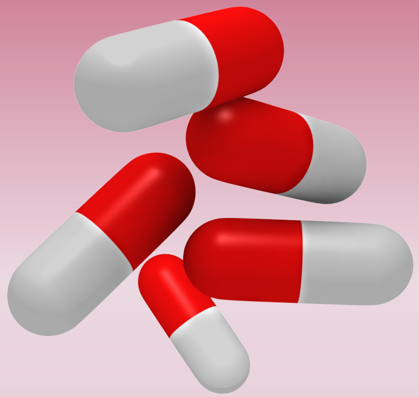

Reducing Pain Without Opioids

Pain Medication

You have received a prescription for Acetaminophen (Tylenol) and Naproxen (Aleve, Naprosyn). We recommend these as **"FIRST STEP"** pain medications.

- Acetaminophen can be taken every 4 hours
- Naproxen can be taken every 12 hours for pain.
- For the first week, take both of these medications on a regular basis even when experiencing minimal pain in an effort to stop post-operative pain before it starts.
- Only in cases where pain persists 1-2 hours after use of Acetaminophen and Naproxen should the opioid prescription (Morphine) be used.

Opioids are intended to improve your pain enough so that you are able to do your day to day activities, but **not reduce your pain to zero.**

The Risk of Addiction

Many people have used opioids without problems. However, serious problems, including overdose and addiction, have happened. It is important to follow the instruction on the prescription and use the lowest possible dose for the shortest possible time, and to be aware of signs of side effects or dependence.

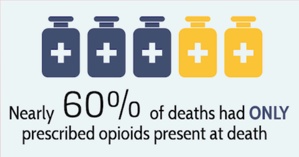

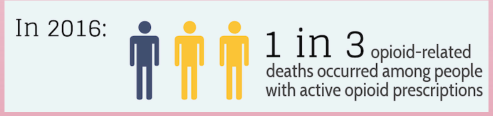

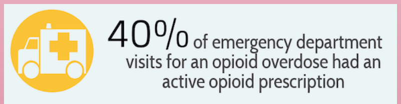

Courtesy of Gomes et al (2018). The BMJ, *362*, k3207.

Managing Opioid Use

Pain management after surgery is about reducing pain so that you can return to normal activities. The goal is to not hit zero on the pain scale, but to avoid levels 8 and above.

Only consider opioid use when your pain levels are more than Severe (level 8).

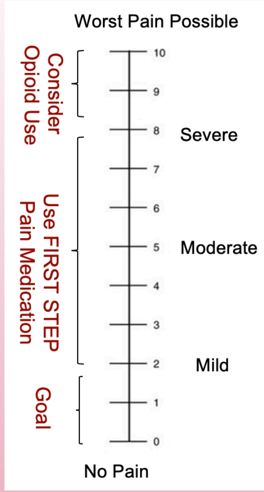

Potential Side Effects of Opioid Use

Nausea, Constipation, Dizziness, Drowsiness, Reduced Physical or Mental Abilities, Depression, Respiratory Issues, Reduced Blood Pressure, Heart Palpitations, Irregular Heartbeat, Problems Sleeping, Including Sleep Apnea, Vision Problems. Other underlying health issues may put you at higher risk or worsen the potential side effects.
